# Liquid Perfluorodecalin Application for In Situ Extraction and Enhanced Naphthoquinones Production in *Arnebia euchroma* Cell Suspension Cultures

**DOI:** 10.1007/s12010-013-0701-5

**Published:** 2014-01-14

**Authors:** Katarzyna Sykłowska-Baranek, Maciej Pilarek, Michał Cichosz, Agnieszka Pietrosiuk

**Affiliations:** 1 Department of Biology and Pharmaceutical Botany, Faculty of Pharmacy, Medical University of Warsaw, Banacha 1, 02-097 Warsaw, Poland; 2 Biotechnology and Bioprocess Engineering Division, Faculty of Chemical and Process Engineering, Warsaw University of Technology, Waryńskiego 1, 00-645 Warsaw, Poland

**Keywords:** Alkannin/shikonin, *Arnebia euchroma*, In situ extraction, Liquid perfluorochemical

## Abstract

Suspension cultures of *Arnebia euchroma* supported with liquid perfluorodecalin (PFD) degassed, aerated, or ethylene-saturated were investigated as a novel in situ extraction system for enhanced alkannin/shikonin production. Simultaneously, the effect of PFD applied as the liquid gas carrier on the growth of *A. euchroma* biomass was studied. The similar dry (4-fold) and fresh (7-fold) biomass increase was observed in the control (without PFD addition) and supplemented with PFD-degassed or PFD-aerated cultures while PFD-ethylene application impeded cell growth. The highest total of alkannin/shikonin production (23.23 mg flask^−1^) was observed when PFD-aerated has been used and it resulted in about 50 % higher yield of alkannin/shikonin compared with the control culture. Chiral HPLC analysis revealed that in cultures supported with PFD, both alkannin and shikonin were produced. Their mutual ratio varied depending on culture conditions, and the accumulation of alkannin prevailed under almost all culture conditions. PFD has proved to be exceptionally efficient and cell-safe solvent for the in situ extraction of naphthoquinone red pigments without exerting any detrimental effects on cell growth. Extracellularly secreted red naphthoquinones were easily dissolved and extracted from the PFD phase, which can be regenerated and reused (e.g., in continuous culture system).

## Introduction

Synthetic liquid perfluorochemicals (synonym: perfluorocarbons, PFCs) are mainly used as liquid gas carriers, which dissolve gases according to the Henry's Law (i.e., the gas transfer rate into PFCs increases linearly with the partial pressure of a component in the gaseous phase) [1–4]. The lack of chemical bonds between gas and PFC also allows the efficient release of dissolved gas, e.g., into a contacting phase of the culture medium. Importantly, PFCs are immiscible with aqueous media and they create a separate phase (i.e., perfluorinated phase) below, i.e., on the bottom of a culture flask/vessel, of the aqueous phase. The lack of toxicity and negative side effects of PFCs on living cells was confirmed by in vitro experiments and also in clinical investigations [4–10]. Until now, the applicability of PFCs in plant cell or tissue cultures has been rather limited. The beneficial effect of PFCs on protoplasts division capabilities, subsequent plant regeneration, and morphogenesis predominantly has been reported or reviewed [9, 10]. More recently, the positive effect of aerated-PFD on the biomass increases, as well as on the extension of the exponential growth phase for *Nicotiana tabacum* BY-2 suspended cultures has been reported [11]. It has also been speculated that PFCs may also be exploited in plant cell systems as scavengers of toxic gaseous by-products, such as ethylene [9–11].

*Arnebia euchroma* (Royle) Johnst. (Boraginaceae), an abundant source of alkannin/shikonin, naphthoquinone-type red pigments used in traditional Chinese medicine since ancient times, is mainly distributed in the western Himalayan region and has been recently reported to be critically endangered due to excessive harvesting for medicinal and commercial purposes [12]. Alkannin/shikonin, as enantiomers, have demonstrated numerous biological activities including strong wound healing, antimicrobial, anti-inflammatory, antioxidant, anticancer, and antithrombotic effects. They are also used as a food and textile colorant [13, 14]. Cell suspension cultures of *A. euchroma* have been successfully established and showed the ability for producing considerable amounts of shikonin and its derivatives. Recent chemical investigations of extracts from in vitro cultured *A. euchroma* biomass revealed the presence of acetylshikonin, alkannin, and their derivatives demonstrating anticancer and antimicrobial activities [15–17].

So far, the enhancement of red pigments production in *A. euchroma* in vitro cultures was achieved by media supplementation with elicitor, precursor or rare elements, modification of medium composition, and inoculum/medium ratio also combined with simultaneous in situ extraction of biosynthesized alkannin/shikonin type compounds [18–22]. However, the beneficial effect of in situ extraction performed with various organic solvents on shikonin accumulation was reported, their application to the culture simultaneously inhibited biomass growth [18, 20]. In shoot cultures of *Lithospermum erythrorhizon,* ethylene has been demonstrated to be significantly involved in the efficiency of shikonin biosynthesis [23]. Ethylene or its precursor application to the closed and sealed culture system resulted in the enhanced accumulation of shikonin derivatives in contrast to significantly lower productivity of these compounds noted in well-ventilated Petri dishes cultures or in the presence of ethylene inhibitors soluble in the culture medium.

The aim of our study was to investigate the influence of perfluorodecalin (PFD) as a liquid gas carrier: degassed (i), saturated with air (ii) or ethylene (iii) on biomass growth and alkannin/shikonin production in cell suspension cultures of *A. euchroma*. Our results also reveal the significant feasibility of PFD as a synthetic, cell-safe solvent for in situ extraction of alkannin/shikonin derivatives from culture medium. To our knowledge, this is the first report on using a liquid PFC in a plant cell culture system for alkannin/shikonin production as well as for in situ extraction of plant secondary metabolites and also one of the first reports on the application of gas saturated PFCs in suspension cultures of plant cells in general.

## Materials and Methods

### Cell Suspension Culture

Cell suspension cultures of *Arnebia euchroma* (Royle) Johnst. were established from callus tissue and subcultured into a 250-ml modified Erlenmeyer flasks containing 50 ml of MSA liquid medium as described earlier [21]. The cell suspension cultures were kept at 25 °C in the dark on an INFORS AG TR 250 shaker (Switzerland) at 105 rpm. Every 4 weeks, 1.5 ± 0.05 g of fresh weight (FW) of cell aggregates were subcultured into fresh liquid MSA medium.

### Perfluorinated Gas Carrier

Perfluorodecalin (PFD, C_10_F_18_; ABCR GmbH & Co. KG, Karlsruhe, Germany), the perfluorinated synthetic analog of decalin, was used as a liquid gas carrier and solvent for in situ extraction of pigments. PFD was autoclaved at 121 °C for 20 min to ensure aseptic conditions. Following this 20 ml of PFD (i.e., degassed PFD, aerated-PFD, or ethylene-saturated PFD) was added to the 50 ml of sterile culture medium. To obtain aerated-PFD, it was saturated with atmospheric air for 15 min. according to the procedure described previously [11]. In the case of PFD saturation with 99.95 % ethylene (Sigma-Aldrich), 5 × 15 s saturation intervals with 15 s intensive manual agitation between the next saturation steps have been done. PFD was added to the medium on the day of inoculation. All flasks were closed with aluminum caps tightly squeezed shut. Although many investigations have been conducted, it is still unclear what type of closure gives the best results [24], the aluminum caps to cover flasks have been chosen for routine work in our laboratory as giving good prevention against contamination and water and humidity loss.

### Experimental Procedures

Experiments were carried out in MSA liquid medium as follows: (1) control culture—without any PFD, (2) culture system with non-saturated PFD (PFD-degassed), (3) culture system with aerated-PFD (PFD-aerated), and (4) culture with ethylene-saturated PFD (PFD-ethylene).

All flasks were inoculated with 1.51 ± 0.01 g of fresh weight (FW) of cells from the 4-week old suspension culture. Samples were harvested every week during the 4 weeks that the cultures lasted.

The biomass increase was measured for FW and dry weight (DW). Cells and media from harvested samples were separated using a Büchner funnel. Then, cells were gently pressed on filter paper to remove excess of medium for further weighing. Cells were lyophilized, and their DW was measured.

All experiments were performed in triplicate. All data shown are mean ± standard deviation (SD). The statistical significance between means was assessed using the analysis of variance (ANOVA) and Tukey’s multiple range test. A probability of *p* < 0.05 was considered significant.

### Chemical Analysis

Red naphthoquinones were extracted from the cells, the aqueous phase of culture medium, and the perfluorinated phase of PFD. For obtaining dye fractions, a powdered sample of lyophilized cells was sonicated with n-hexane. The extraction was done for 15 min at 40 °C until the red coloration had faded. Prior extraction phases of medium and PFD were placed into separatory funnel and separated carefully. Afterwards, the aqueous phase was filtered and extracted with n-hexane as described above for cells. The PFD phase was directly washed twice with methanol. Methanolic extracts were combined and evaporated to dryness under reduced pressure. Next, the phase of PFD deprived of red pigments was washed twice with distillated water and reused in subsequent experiments.

All extracts prepared from cells, aqueous and PFD layers were dissolved in methanol and analyzed with a DIONEX HPLC system under the conditions described earlier [25].

In order to determine the total alkannin/shikonin content obtained from suspension cultures, extracts were subjected to alkaline hydrolysis according to the method presented by Boehm et al. [26]. The absorbance was measured at 520 nm on NanoDrop 2000c (Thermo Scientific, USA) spectrophotometer device.

In order to perform chiral analysis of the investigated extracts, the method by Ikeda et al. [27] was applied. The chiral column (250 × 4 mm; 5 μm) Lux Cellulose-3 (Phenomenex, USA) was used, and absorbance was measured at 520 nm. Retention time for shikonin was 10.360 min while for alkannin, 12.481 min. The alkannin standard was purchased in Carl Roth GmBH (Germany) and all other chemicals in Sigma-Aldrich.

## Results and Discussion

### Effect of PFD on Cell Biomass Growth

**Fig. 1:**
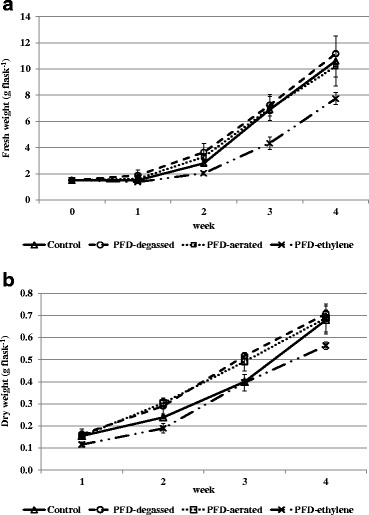
Time course of fresh (**a**) and dry (**b**) biomass accumulation by cell suspension culture of *A. euchroma* carried out in 250-ml flasks containing 50 ml of liquid MSA medium (control) or 50 ml of MSA medium with addition of 20 ml PFD-degassed, PFD-aerated, or PFD-ethylene

The primary aim of our work was to examine the influence of PFD on cell growth of red naphthoquinone pigment producing cell suspension culture of *A. euchroma*. The influence of PFD-degassed, PFD-aerated, and PFD-ethylene compared with the control culture without any PFD has been analyzed. The comparison of cell growth under these culture conditions has been presented in Fig. 1.

There were no significant differences (*p* < 0.05) between fresh biomass increase in control and PFD-degassed or PFD-aerated cultures throughout the whole cultivation period (Fig. 1a). These results could be attributed to the incomplete water removal before cell weighing, and dry weight seems to be a more appropriate parameter to describe cell growth.

The time course of dry biomass increase revealed the distinct and statistically significant differences between the control culture and cultures supplemented with PFD, except the culture with PFD-ethylene. Medium supplementation with PFD-degassed and aerated was favorable for cell growth. The higher dry biomass accumulation in cultures with PFD-degassed and PFD-aerated layers was observed from the beginning till the third week of cultures. But finally, after 4 weeks of culture, the dry biomass increased in PFD-supplemented cultures: degassed and aerated and in the control culture achieved the same level (Fig. 1b). The final fresh and dry biomass increase (expressed as a ratio of final weight to initial weight) in control and PFD-degassed or PFD-aerated cultures amounted to 7-fold and 4-fold, respectively. The cell growth was remarkably diminished by the application of ethylene-saturated PFD, which may be explained by the activity of ethylene as an inhibitor of cell’s mitotic divisions [28].

Cell suspension cultures carried out under the conditions of this experiment did not achieve the stationary phase of growth. Until the end of the experiment, they remained in the exponential growth phase. Previous investigations on the suspension cultures of *A. euchroma* of the same cell line in MSA medium demonstrated the onset of the stationary growth phase on the 21st day of culture, and the final biomass increase was slightly higher than in the current investigation (8-fold) but it was determined on day 35, as the last day of culture [21]. The prolonged exponential growth phase in plant cell suspension cultures as an effect of support of a culture system with PFD was first reported by Pilarek and Szewczyk [11] for *Nicotiana tabacum* cells. The authors postulated that two compared cell lines of *N. tabacum* differ in susceptibility to the presence of the PFD phase in the medium, as the reaction on PFD-aerated in non-genetically modified tobacco cell line was more pronounced than in the BY-2 genetically modified one.

### Effect of PFD on Alkannin/Shikonin and its Derivatives Production

**Fig. 2:**
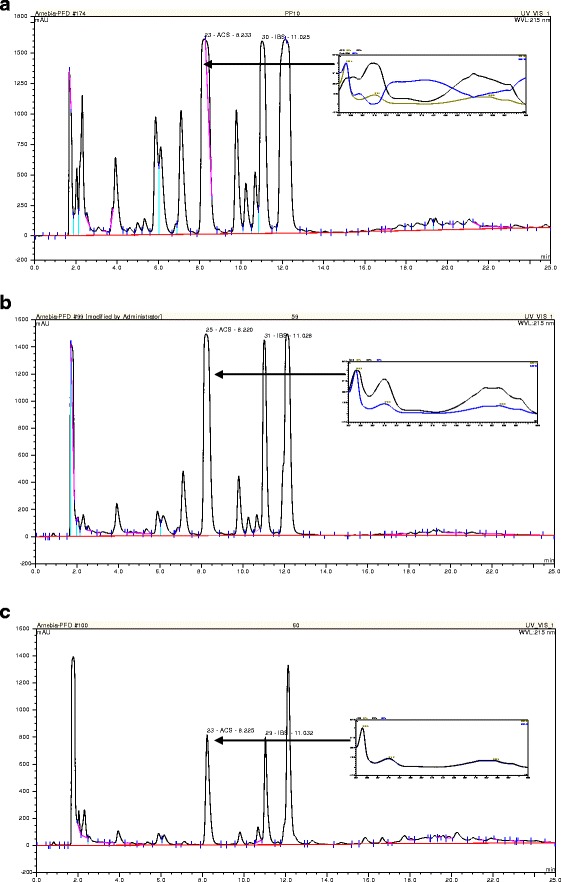
Chromatogram of HPLC separation of extracts prepared form cell suspension cultures of *A. euchroma* carried out in liquid MSA medium supplemented with PFD-aerated for 28 days: **a** cells, **b** culture medium, **c** PFD-aerated phase

First, we implemented the HPLC-DAD analysis to evaluate naphthoquinone pigments yield in suspension cultures of *A. euchroma*. The concentration of alkannin/shikonin and their derivatives was determined in cell and both aqueous and PFD phases of the culture system. The culture system without the PFD phase was considered as a reference culture. In all analyzed extracts, neither alkannin nor shikonin was detected but from three up to eight different alkannin/shikonin derivatives were found irrespectively of culture conditions. The major derivative seemed to be acetylshikonin but other derivatives also occurred in considerable amounts. Because the identification of compounds other than shikonin, alkannin, acetylshikonin, and isobutyrylshikonin was not possible due to the lack of standards, we decided to perform hydrolysis of the investigated extracts. Moreover, acetylshikonin was overlapped by another compound with a UV spectrum different from alkannin/shikonin, which rendered acetylshikonin quantitative estimation impossible (Fig. 2a,b). Finally, chiral analysis of extracts was done.

**Fig. 3:**
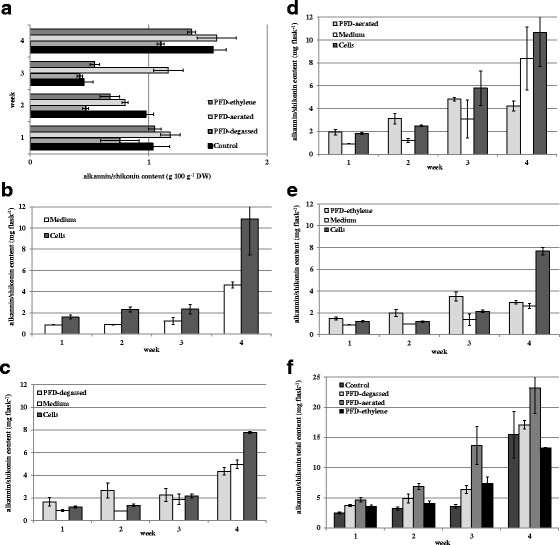
Alkannin/shikonin accumulation in cell suspension cultures of *A. euchroma* carried out in liquid MSA medium: **a** in cell dry biomass (g 100 g^−1^ DW), **b** in control cultures—without PFD, **c** in cultures maintained in the presence of PFD-degassed, **d** in cultures maintained in the presence of PFD-aerated, **e** in cultures maintained in the presence of PFD saturated with ethylene, **f** total content: intracellular + combined extracellular: aqueous and PFD phases

The highest total of alkannin/shikonin content (intracellular, extracellular: combined aqueous and PFD phases) was detected in the suspension culture performed in the presence of PFD-aerated 23.23 mg flask^−1^ (Fig. 3f). Application of PFD-ethylene resulted in the lowest total red pigment production, 13.27 mg flask^−1^, which could be attributed to the lowest cell biomass growth.

As is demonstrated in Fig. 3a, red pigment accumulation in cells (g 100 g^−1^ DW) did not coincide with biomass accumulation (Fig. 1). The highest alkannin/shikonin content was detected in cells from the control culture and the culture supplemented with PFD-aerated 1.54 and 1.57 g 100 g^−1^ DW, respectively. In cells from cultures carried out in the presence of PFD-degassed and PFD-ethylene, the red naphthoquinone content was 1.10 and 1.36 g 100 g^−1^ DW, respectively.

The final total yield of red pigments, calculated per flask, in cultures supplemented with PFD-degassed and PFD-aerated was significantly higher, 10 and 50 %, respectively, compared with the control culture. In almost all cultures supplemented with PFD, except PFD-ethylene (Fig. 3e), the higher extracellular amounts of red compounds rather than intracellular were detected (Fig. 3c,d).

In the control culture, the level of cell that associated alkannin/shikonin was 2-fold higher than the extracellular one (Fig. 3b). The opposite effect was observed in cultures supplemented with PFD-degassed and PFD-aerated, where alkannin/shikonin was mostly accumulated in aqueous and PFD phases. In the third week, the concentration of red pigments in the aqueous phases exceeded the amount detected in PFD ones (Fig. 3c,d). In the culture with PFD-ethylene, such a relation was extended on the whole of the culture time (Fig. 3e).

The culture system supplemented with PFD-ethylene resulted in the lowest biomass accumulation combined with the lowest pigment production. Our results have been in opposition to results described by Touno et al. [21] where the authors presented stimulatory effect of ethylene applied to the shoot culture of *L. erythrorhizon* on shikonin accumulation without inhibition of shoot growth. Moreover, the addition of ethylene inhibitors: silver ion or aminoethoxyvinylglycine (AVG) decreased its production.

The HPLC chiral analysis of all extracts showed that in all cases, alkannin and shikonin derivatives were present. However, their mutual proportions depended on the culture conditions. In the control culture, almost 8-fold higher amounts of alkannin in comparison with shikonin were detected in cells while in the medium shikonin was the predominant enantiomer. In the cultures supplemented with PFD-aerated and PFD-ethylene alkannin was prevailing over shikonin in all types of extracts. Moreover, in cells growing under the influence of PFD-ethylene, alkannin content was 26-fold higher than shikonin. In the culture maintained in the medium supplemented with PFD-degassed, the equal amounts of alkannin and shikonin were noted in cells and the PFD phase. But in the aqueous phase, alkannin 6-fold exceeded shikonin content.

In previous investigations on suspension cultures of the same cell line of *A. euchroma*, Syklowska-Baranek et al. [21] have demonstrated that considerably higher amounts of shikonin derivatives have been accumulated in cells than those excreted to the culture medium. The highest total (i.e., cell associated and extracellular) yield of shikonin derivatives: acetylshikonin and isobutyrylshikonin amounted to 9.5 mg flask^−1^.

The appreciable amount of red pigments was permeated into PFD phases augmenting their final productivity under conditions of our experiments. It has been previously demonstrated that in situ extraction exerts adventitious effect on plant secondary metabolites including shikonin type naphthoquinones. Kumar et al. [20] obtained 49 and 73 % higher shikonin production in cell suspension cultures of *A. euchroma* by application as in situ solvents liquid light (552.5 mg L^−1^) and heavy paraffin (637.2 mg L^−1^), respectively. Nevertheless, these organic solvents significantly inhibited cell growth, which was attributed to the seriously reduced gas transfer in the paraffin supported culture system.

Another type of organic solvent, n-hexadecane, was used simultaneously with fungal elicitor in cell suspension culture of *A. euchroma*, and the increase in product yield (245.7 mg L^−1^) was 6.15-fold higher than for the control untreated culture [18]. A similar synergetic effect of fungal elicitor treatment together with in situ extraction with n-hexadecane on shikonin production was reported earlier [29] for *L. erythrorhizon* suspension culture. Shikonin production (60 mg L^−1^) was 24-fold higher compared with the control culture.

In situ product removal strategy was also successfully implemented in hairy root of *L. erythrorhizon*. Recovery of shikonin (over 99 %) was accomplished with the highest efficiency by application of XAD-2 polymeric adsorbent with the increase in shikonin yield up to 1.5 g L^−1^ [30] or n-hexadecane (over 95 %) [31] when shikonin production increased up to 120.6 g L^−1^ as a result of in situ extraction. In the latter paper, the authors reported the beneficial influence of the organic solvent on hairy roots growth. The organic solvents and polymeric resins were postulated to diminish product feedback inhibition by removing shikonin and other compounds, which could be deleterious for cell and tissue growth.

In our studies presented in the current work, the release of red pigments into the PFD phase, which varied from 18 to 55 %, depends on treatment (Fig. 3) with their complete (100 %) recovery from the PFD phase.

**Fig. 4:**
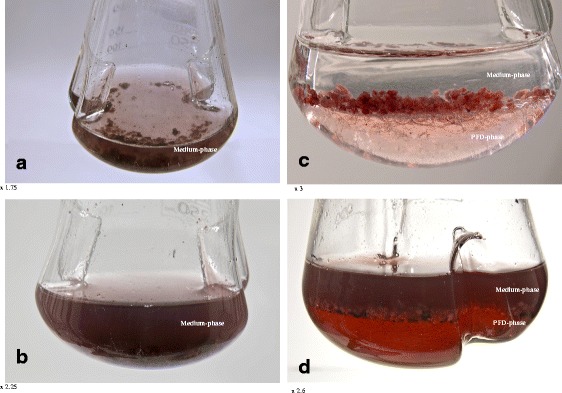
*Arnebia euchroma* cell suspension culture carried out in liquid MSA medium: **a** control culture—day of inoculation, **b** control culture—last day of cultivation, **c** culture supplemented with PFD-aerated—day of inoculation, **d** culture supplemented with PFD-aerated—last day of cultivation

Summarizing, this is the first research on the application of liquid PFC in plant cell culture to enhance secondary metabolites production and also the first report presenting the feasibility of liquid PFCs to enhance productivity by in situ recovery extraction with stimulation of biomass accumulation. Our results revealed the enormous properties of PFD to remove excreted secondary metabolites with no detrimental effect on cell growth (Fig. 4). Moreover, application of PFD-aerated resulted in a significant improvement of total alkannin/shikonin productivity. The recovery of the PFD from aqueous culture medium was simple and allowed it to be regenerated, i.e., saturated with the given gas and then re-used.
